# Collagenase-Induced Patellar Tendinopathy with Neovascularization: First Results towards a Piglet Model of Musculoskeletal Embolization

**DOI:** 10.3390/biomedicines10010002

**Published:** 2021-12-21

**Authors:** Julien Ghelfi, Marylène Bacle, Olivier Stephanov, Hélène de Forges, Ian Soulairol, Pascal Roger, Gilbert R. Ferretti, Jean-Paul Beregi, Julien Frandon

**Affiliations:** 1 Service de Radiologie Diagnostique et Interventionnelle, CHU Grenoble Alpes, 38043 Grenoble, France; jghelfi@chu-grenoble.fr (J.G.); gferretti@chu-grenoble.fr (G.R.F.); 2 Department of Medical Imaging, Nîmes University Hospital, University of Montpellier, Medical Imaging Group Nîmes, 30000 Nimes, France; helene.deforges@chu-nimes.fr (H.d.F.); jean.paul.beregi@chu-nimes.fr (J.-P.B.); 3 Faculty of Medicine, Montpellier Nîmes University, RAM-PTNIM, 30000 Nimes, France; marylene.bacle@umontpellier.fr; 4 Anatomopathology Department, Grenoble University Hospital, 38043 Grenoble, France; ostephanov@chu-grenoble.fr; 5 Department of Pharmacy, Nîmes University Hospital, 30000 Nimes, France; ian.SOULAIROL@chu-nimes.fr; 6 ICGM, University of Montpellier, CNRS, ENSCM, 34090 Montpellier, France; 7 Anatomopathology Department, Nimes University Hospital, University of Montpellier, 30000 Nimes, France; pascal.roger@chu-nimes.fr

**Keywords:** patellar tendon, tendinopathy, neovascularization, embolization, animal model

## Abstract

Background: Therapeutic strategies targeting neovessels responsible for musculoskeletal chronic pain have emerged, including neovessels embolization. Our study aimed to develop a large animal model of patellar tendinopathy with neovascularization. Methods: Nine 3-month-old male piglets (18 patellar tendons) received percutaneous injections of increasing doses of collagenase (0 to 50 mg) at day 0 (D0). Tendinopathy was evaluated by ultrasound (D7 and D14). Neovascularization was evaluated visually and on angiographies. Bonar score was used for histological analysis (D14). Correlations were evaluated using Spearman’s rank (Rs) test. Results: Research protocol was well tolerated. All tendons were enlarged with a median increase of 31.58% [25–40.28] at D7 (*p* = 0.244) at D7 and 57.52% [48.41–91.45] at D14 (*p* = 0.065). Tendons with collagenase injection had more hypoechoic changes, with one tendon rupture (*p* = 0.012). Neovascularization was reported above 5 mg collagenase (*p* < 0.01) at D7 and D14 with dose-related neovessels induction (Rs = 0.8, *p* < 0.001). The Bonar score increased above 5 mg collagenase, correlated with the dose (Rs = 0.666, *p* = 0.003). Conclusions: The study shows the feasibility, safety and reproducibility of this new large animal model of patellar tendinopathy with neovascularization after collagenase injection. It will allow studying new treatments on direct embolization of neovessels by endovascular approach.

## 1. Introduction

Tendinopathy is a common and significant clinical problem. This disabling condition mostly affects active young and middle-aged people, and has a significant impact on their quality of life [1], on both personal and professional activities. The major symptom of tendinopathy is chronic pain [2]. Athletes and sportsmen/women are especially impacted with 14% reporting chronic patellar tendinopathy [3]. Indeed, up to 53% of athletes with patellar tendinopathy had to quit their sports career because of their knee problem [4].

The therapeutic strategy begins with conservative management options [5] such as physical therapy, activity modification, icing and administration of nonsteroidal anti-inflammatory drugs [6], peri-tendinous injections of corticosteroids [7,8], and platelet-rich plasma intra-tendinous injections [9]. Although such therapeutic strategies reported good success rates, approximately 10% of patients are unresponsive and are proposed surgical treatments including open or arthroscopic procedures [3].

Tendinopathy leads to neoangiogenesis which was shown to be associated with nerve fibers development [8,10]. Studies have shown these neovessels and neonerves account for a major source of tendon pain [11,12]. Alternative techniques targeting these neovessels have recently emerged for the treatment of tendinopathy: ultrasound-guided sclerosis has shown pain reduction [13,14], electrocoagulation of neovessels for chronic patellar tendinopathy was also described [15]. Recently, intra vascular embolization of these neovessels in patients with knee osteoarthritis was described by Okuno et al. with a high clinical success and up to 80% of persistent pain reduction at 3 years [16,17]. The same team also reported a good efficacy on few cases of patellar tendinopathy [18].

New strategies against chronic pain could aim at limiting neoangiogenesis and neonerves development in tendinopathy. It thus has become essential to better understand these mechanisms. Small animal models of tendinopathy have been developed and successfully used in few studies [19,20]. Some of them specifically studied the development of neovessels [21,22,23] that seem to appear between 3 and 15 days after tendinopathy induction [24], with a peak at day 7 (D7) in rats. Boesen et al. demonstrated that treatment of tendinopathy targeting neovessels could be extrapolated from human studies to animals with the efficacy of sclerosing neovessels in Achille’s tendinopathy in horses [25]. Furthermore, Taguchi et al. showed embolization of these neovessels was efficient in frozen shoulders in rats [19].

A large animal model of tendinopathy with neovascularization and possibility to perform embolization is mandatory to better understand the mechanism of this promising new therapeutic strategy. The present study aimed to evaluate the feasibility of a chemically-induced piglet model of patellar tendinopathy with focus on neovessels.

## 2. Materials and Methods

### 2.1. Animal Model

The study was performed with authorization of the local government animal rights protection authorities (Languedoc-Roussillon No 36, ID number Nr 2018011916269335 #13156 v3) in accordance with the National Institute of Health guidelines for the use of laboratory animals.

**Figure 1:**
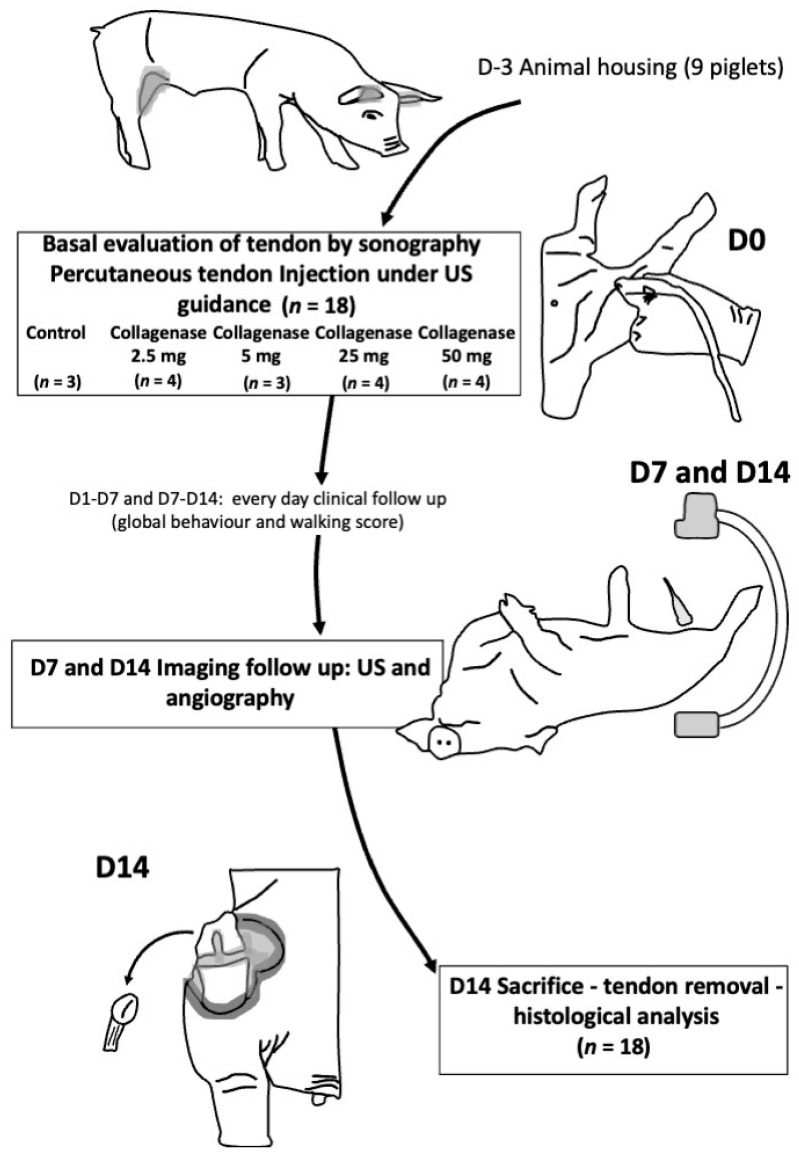
Flowchart of the study. US: ultrasound; D: day.

Nine 3-month old male piglets, weighting around 20–30 kg, were used. They were housed by groups of 3; three sessions were performed after 5 days housing for piglet acclimation. The study protocol is summarized in Figure 1.

For tendon injection and basal ultrasound evaluation of the tendon at inclusion, piglets were sedated with 1 mg Midazolam, 10 mg ketamine and 5 mg azaperone per kilogram of body weight. For angiography evaluation and ultrasound follow-up at D7 and D14, piglets underwent general anesthesia, after overnight fasting with free access to water. They were premedicated with intramuscular injection of 10 mg/kg ketamine, 1 mg/kg midazolam and 0.05 mg/kg atropine. Analgesia was provided by injection of 10 μg sufentanil before the procedure. Anesthesia was induced with a propofol bolus (4 mg/kg) via an ear vein and was then maintained with propofol (10 mg/kg/h). The animals were ventilated after intubation (Datex-Ohmeda Aestiva 3000 ventilator). At D14, euthanasia was performed after the angiographic procedure by intravenous injection of 60 mg/kg phenobarbital.

### 2.2. Induction of Patellar Tendinopathy

Under ultrasound guidance (Vivid 7, GE Healthcare, Marlborough, MA, USA), collagenase type I (sigma-aldrich, St Louis, MO, USA) was percutaneously injected directly inside the patellar tendon at its mid part with a 0.5 mL syringe and a 29G needle (Terumo, Tokyo, Japan). The amount of collagenase injected was allocated as follows: 1 pig (2 tendons) was not injected (controls); 2 pigs received a 2.5-mg dose per tendon (4 tendons); 1 pig was injected a 5-mg dose in one tendon and the second was not injected (control); 1 pig was injected 5 mg collagenase in both tendons (2 tendons), 2 pigs 25 mg in both tendons (4 tendons), and 2 pigs 50 mg in both tendons (4 tendons). Day 0 was set-up at the intra-tendinous injection.

### 2.3. Clinical Assessment

The piglets were not restrained and allowed to walk within their collective enclosure during the following 2 weeks. They were daily monitored by 2 zoo-technicians with more than 5 years of experience. They evaluated appetite (/2), activity (/4), reactivity (/2) and walking ability (/3) using a home-made global behavior scale on 11 points. The specific walking score was as follows: 0: reluctance to move around and bear weight on one leg; 1: marked lameness on at least one limb; 2: easy movements but small lameness on at least one leg; 3: easy movements and comfort on 4 legs. Piglets were weighed every day at meal time. A loss of weight more than 20% of the initial body weight or a deterioration of the general condition with total immobility during more than 24 h were decided as criteria for euthanasia.

### 2.4. Ultrasound Exploration

Exploration was performed at D0, D7 and D14 with the same Vivid 7 (GE Healthcare) and aimed at looking for tendon changes: increased thickness at mid portion, hypoechoic heterogeneity, hyper vascularization with power Doppler, and rupture.

### 2.5. Angiography Exploration

Endovascular explorations were performed at D7 and D14 by two interventional radiologists (JF, 10-year experience and JG, 5-year experience) on a Fluorostar III (GE Healthcare). A left carotid arterial access was performed under ultrasound guidance using the Seldinger technique. A 5F introducer sheath was set and both superficial femoral arteries were catheterized with a 5F catheter. Selective angiography in both genicular arteries was performed with contrast media (OMNIPAQUE 350, GE Healthcare) and a 2.0 Fr microcatheter with front and side views focus on the patellar tendon area were acquired. Digital subtraction angiography images were analyzed in a random order three months after procedure by the two radiologists, blinded to injection protocol. They visually characterized neovascularization in consensus as none, mild or important.

### 2.6. Histological Analyses

Surgical dissection of both knees was performed immediately after sacrifice and allowed removing the patellar tendons. Tendons were fixed with 4% formaldehyde and cut longitudinally and transversally, then embedded in paraffin. Histological sections were made with a microtome (3-µm thickness slices) and mounted on glass slides for staining (HES, Alcian blue) and histopathological examination. The histological examination was performed in a blinded manner by a single pathologist (OS) with 5 years’ experience on an Olympus BX51 microscope. Tendinopathic tissue changes were characterized with the Bonar score [26,27,28] and its 5 characteristics: collagen fiber arrangement, cell morphology, cellularity, vascularization and accumulation of ground substance. Each characteristic was graded from 0 (normal tendon tissue) to 3 (advanced changes). We focused on the vascularity subclass index.

### 2.7. Statistical Analysis

Statistical analysis was performed using Biostatgv (http://marne.u707.jussieu.fr/biostatgv; accessed on 10 May 2021). Quantitative variables are presented using medians and interquartile ranges (1st–3rd). Qualitative variables are presented with numbers. Values were compared using the Kruskal–Wallis test with a significance level set-up at *p* < 0.05. Spearman’s rank analyses were performed to evaluate the correlation between the Bonar score, neovascularization on angiography and histology, and the dose of collagenase injected.

## 3. Results

### 3.1. Development of Tendinopathy Model

The study was performed on nine piglets of median weight 30.2 kgs [IQR range: 22.5–33.1]. On ultrasound exploration, all patellar tendons were enlarged: the median increase was of 32% (25–40.28) at D7 and of 58% (48.41–91.45) at D14, without any difference between the groups (Table 1). Hypoechoic changes appeared above 5 mg collagenase, with a significant difference between groups (*p* = 0.012). One case of tendon rupture was reported in the 25 mg collagenase group.

**Figure 2:**
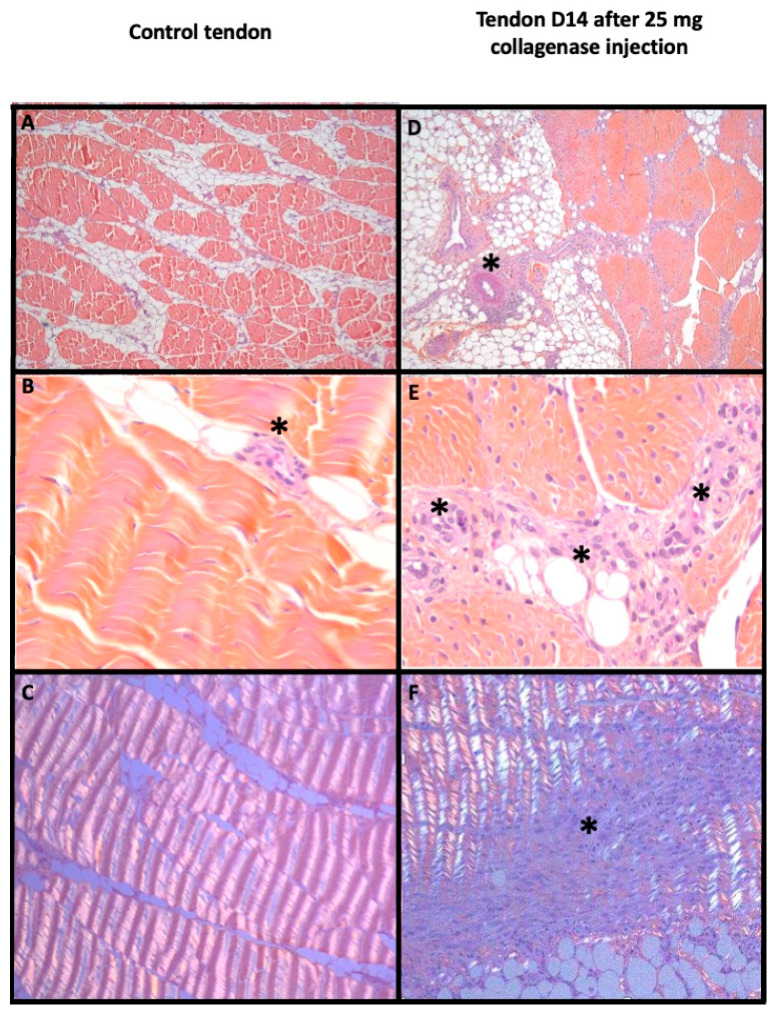
Histological evaluation of a control tendon (left) and a pathological tendon 14 days after injection of 25 mg of collagenase (right). Patellar tendon with HES stain ×50 (**A**,**D**), ×400 (**B**,**E**) magnification and ×100 with polarization (**C**,**F**). In the control tendon, (**A**,**B**) show regular bundles of small spindle fibroblasts in wavy collagen with few small capillaries between bundles (black asterisk); (**C**) depicted the regular and wavy aspect in polarization. In the pathological patellar tendon, fibrinoid necrosis appeared in peri-tendinous artery (black asterisk) in (**E**) with loss of demarcation of fibers bundles, plump fibroblasts and numerous clusters of capillaries (black asterisk) in (**D**). (**F**) showed focal distortion of wavy appearance in polarization (black asterisk).

On histology, a significant correlation between the amount of collagenase injected and the Bonar score was found (Rs = 0.666, *p* = 0.003). Tendon modification appeared after injection of collagenase with a Bonar score significantly increasing from 2/15 (control) to 13/15 for 25 and 50 mg (*p* = 0.024, Table 2, Figure 2).

### 3.2. Neovascularization Evaluation

**Figure 3:**
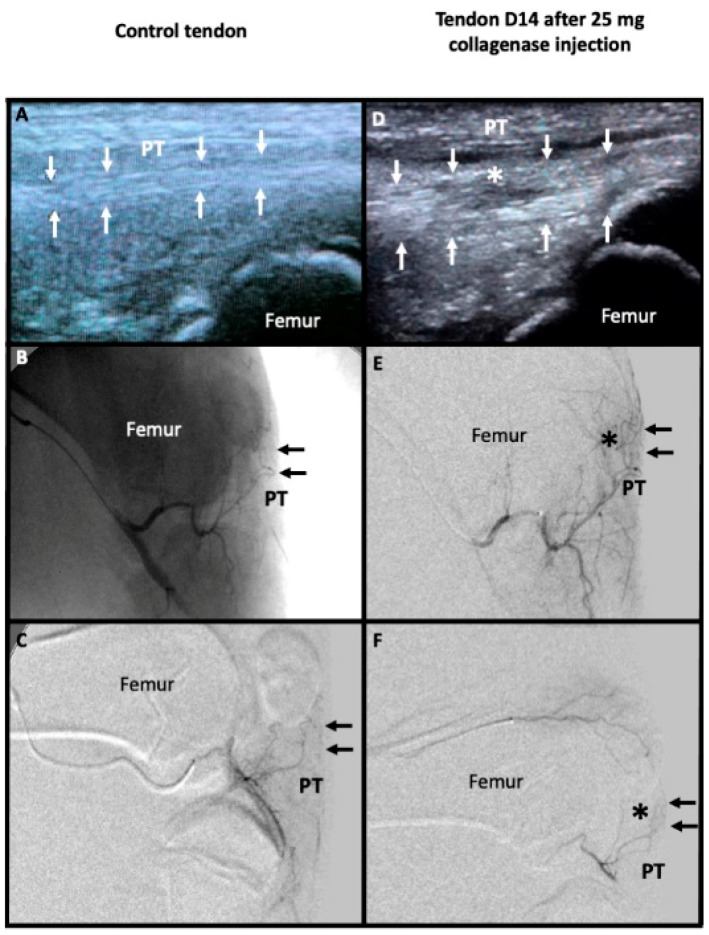
Imaging evaluation between a control tendon (left) and a pathological tendon 14 days after injection of 25 mg of collagenase (right). Sagittal ultrasound acquisition show the regular hyperechoic fibers of the control patellar tendon (PT) framed by small white arrows in (**A**). Microcatheter was placed inside the main genicular artery to perform unsubstracted angiography (without bone substraction which is still visible) in (**B**) and digital substraction angiography showing only the vessels like in (**C**). PT was marked with small black arrows (**B**,**C**,**E**,**F**). There were no abnormal microvessels in projection of PT in the control tendon (**B**,**C**). The pathological tendon showed a marked thickening framed by small white arrows with hypoechoic change in its mid portion (white asterisk) in (**D**). Angiography in the main genicular artery (**E**) and in the superior genicular artery (**F**) showed abnormal microvessels in projection of PT (black asterisk).

No neovessels were visible with ultrasound except for the case which reported tendon rupture. Angiographic findings were similar at D7 and D14 for all tendons. Collagenase dose-related neovessels formation was reported (Rs = 0.8, *p* < 0.001, Table 2). Neovascularization was more frequently quoted “important” after injection of 5 mg collagenase or more, which was significant (*p* = 0.009, Figure 3).

On histology, the vascularity subclass index of the Bonar score was higher after collagenase injection than in controls, increasing from 1/3 (controls) to 3/3 (collagenase), without significant difference (*p* = 0.054, Table 2).

### 3.3. Efficiency of the Model Obtained

The baseline global behavior score was of 11 for all piglets (Table 3). Most piglets showed the same global behavior at D7 and at D14 than at baseline. Two piglets injected with 2.5 mg collagenase showed a poorer box behavior score at D1, following collagenase injection, which returned to normal as soon as D2. The injured hind limbs were slightly stressed and the activity of the animals was not impaired after collagenase injection. There were no abnormal findings or signs of knee tendon.

All angiographic explorations were performed without any complication and all genicular arteries were accessible with the 2.0 Fr microcatheter. Collagenase injection allowed rapid development of tendinopathy on ultrasound and angiography. Indeed, signs of tendinopathy were visible at D7, with no significant difference between D7 and D14.

Regarding clinical tendinopathy symptoms, collagenase injection had no effect on the walking score at D7 and D14, whatever the dose injected (Table 3).

## 4. Discussion

This study showed that injection under ultrasound guidance of type 1 collagenase inside the patellar tendon allowed the generation of a tendinopathy model with neovessels in piglets. The model was well tolerated by pigs without any reported animal suffering sign. From day 7 after injection, tendinopathy with tendon modification on ultrasound was observed, together with neovascularization visible on angiography and confirmed by histology at day 14. Tendon modification was correlated with the amount of collagenase injected.

Chemically-induced models in animals are easier to put in place, more reproducible and with short term efficacy [29]. Collagenase-induced tendinopathy has been reported in rat [24], rabbit [21] and equine [25] Achilles’ tendons. In this study, the patellar tendon was preferred to the Achilles’ tendon for tendon access, vessel size and endovascular accessibility of femoral instead of tibial arteries.

At day 7 and day 14 imaging evaluation, all piglets presented an enlargement of tendon on ultrasound without significant difference in piglets injected with collagenase. This may be explained by the tendon rupture observed in the 25 mg group, responsible for underestimate size measure. Tendon degeneration was evaluated using the revisited Bonar score for histological analysis [26] to stay close with clinical practice [9,30]. The Bonar score was correlated with the collagenase dose injected in accordance with a previous study in rats [24].

Piglets show a vascular anatomy close to that of humans and are large enough to be explored using imaging devices used in human clinical practice, contrary to small animal models. They have been widely used as models for endovascular studies [8,19,20]. No neovascularization was seen on power ultrasound, although it was reported on angiography, which may be due to a limited sonographer sensibility. Neovessels growth was described between 3 and 15 days after injection in a rat Achilles’ tendinopathy model [24], and a peak of neovascularization at day 7 in another rat model of patellar tendinopathy [31]. In this study, similar neovascularization was observed on angiographies at day 7 and day 14 with collagenase dose-related neovessels induction.

The vascularity subclass index also increased after injection. This is concordant with the results of Taguchi et al. who showed increasing number of neovessels in frozen shoulders in rats [19]. However, no statistical difference was reported in our study. This could be explained by the presence of neovessels in all control tendons with a vascularity subclass score of 1 like it was already described in normal patellar tendon in rats [31]. These results should be taken with caution. Indeed, a recent study has discussed the vascularity subclass index of the Bonar score which may not be well adapted to tendinopathy and could be revised [32]. Furthermore, neovascularization in tendinopathy is a complex process implicated in wound healing. Correlation between neovascularization intensity and tendinopathy symptomatology has not been demonstrated [33].

Our study has developed a tendinopathy model with neoangiogenesis, with possibility of histologic evaluation and follow-up of the Bonar score. This new model may now be useful to assess new popular treatments such as platelet-rich plasma (PRP) treatments and their effects on tendon stem cells. Indeed, these new therapies are yet evaluated either in vitro [34] or in clinics [35]. Preclinical data (anapathological and histological data) are lacking to better understand tendinopathy mechanisms.

This pig model presented some limitations. The main limitation was the absence of clinical symptomatology of tendinopathy after collagenase injection. If confirmed in further studies, this model will not allow studying clinical responses to new therapies targeting neovessels. On the other hand, response can be evaluated on angiography or on histology after piglet euthanasia. Another limitation was that neovessels were evaluated visually on angiographies. It could be interesting to use a quantitative analysis with angiography analyses dedicated software [36] or other imaging tracers of vascularization such as USPIO in MRI [31]. These neovessels could only be seen on angiography and not using color Doppler ultrasonography. This will not allow the study of the sclerosing effect of percutaneous injection as it was previously published on the treatment of tendinopathy in horses [25]. Angiography showed capillaries blush close to human neovascularization described by Okuno in his landmark studies on neovessels embolization for osteo-articular and tendinous chronic pain [17,18]. These neovessels were accessible by catheterism and offer the possibility to perform embolization to better understand the mechanism of this promising new therapeutic strategy. Last, although piglets do not have the same ethical limits than other large animal models such as horses or dogs, they are more expensive (price and housing costs) than small animal models such as rats or mice.

## 5. Conclusions

This study confirmed the feasibility of a model of tendinopathy in piglets, with collagenase-induced modification of the tendon, neovascularization and neovessels formation. It was safe and well tolerated by piglets allowing further research on the embolization of these neovessels to study the effect of neovessel thrombosis on tendon architecture and better understand the efficacy of embolization in chronic tendinopathy pain relief.

## Figures and Tables

**Table 1:** Ultrasound evaluation of patellar tendon (surface and morphological changes) at D0 and at D7 and D14 after direct injection of collagenase inside the tendon.

	Tendon Surface at Baseline (cm^2^)		Increased Tendon Surface Compared to Surface at Baseline, % [IQR range]				Tendon Lesions (n)				
	Median [Range]	*p*-Value	D7	*p*-Value	D14	*p*-Value	None	Hypoechoic Change	Neovas-Cularization	Tendon Rupture	*p*-Value
Control\(*n* = 3)	0.19 (0.19–0.19)	0.244	32 (29–31)	0.167	53 (50–56)	0.065	3	0	0	0	0.012
2.5 mg Collagenase (*n* = 4)	0.18 (0.18–0.19)		37 (16–67)		40 (34–47)		4	0	0	0	
5 mg Collagenase (*n* = 3)	0.27 (0.24–0.27)		25 (16–46)		89 (77–96)		1	2	0	0	
25 mg Collagenase (*n* = 4)	0.30 (0.26–0.33)		37 (20–44)		54 (45–60)		0	3	1	1	
50 mg Collagenase (*n* = 4)	0.18 (0.15–0.21)		29 (26–37)		104 (92–124)		1	3	0	0	

**Table 2:** Angiographic neovascularization evaluation on patellar tendon at D7 and D14 after model induction and Bonar score after sacrifice.

	Angiographic Neovascularisation (Visual Evaluation)										BONAR Score at Day 14					
	Day 7					Day 14										
	None (N)	Mild (N)	Important (N)	*p*-Value	Rs *p*	None (N)	Mild (N)	Important (N)	*p*-Value	Rs *p*	Global Score Median [IQR]	*p*-Value	Rs *p*	Vascularity Subclass Median [IQR]	*p*-Value	Rs *p*
Control (*n* = 3)	3	0	0	0.009	0.82 <0.001	3	0	0	0.008	0.791 <0.001	2.0 (2.0–2.0)	0.024	0.666 0.003	1.0 (1.0–1.0)	0.054	0.373 0.127
2.5 mg Collagenase (*n* = 4)	2	2	0			2	2	0			2.5 (2.0–4.5)			1.0 (1.0–1.5)		
5 mg Collagenase (*n* = 3)	0	2	1			0	1	2			6.0 (4.0–8.5)			2.0 (1.5–2.5)		
25 mg Collagenase (*n* = 4)	0	0	4			0	0	4			7.5 (5.8–10.0)			1.5 (1.0–2.3)		
50 mg Collagenase (*n* = 4)	0	1	3			0	1	3			9.0 (6.8–10.8)			2.0 (1.8–2.0)		

**Table 3:** Clinical follow up evaluating the global behavior with a 11-point scale, a focus on the walking ability on a 3 point scale and the weight gain. P: piglet; R: right; L: left.

Piglets (Amount of Collagenase Injected; R and L, in mg)	Global Behavior					Walking Score					Weight (kg)			
	Baseline	D1	D7	D14	*p*-Value	Baseline	D1	D7	D14	*p*-Value	Baseline	D7 Weight Gain	D14 Weight Gain	*p*-Value
P1 (R:0, L:0)	11	11	11	11	1	3	3	3	3	1	33.7	6%	17%	0.956
P2 (R:0, L:2.5)	11	9	11	11		3	1	3	3		30.2	2%	19%	
P3 (R:2.5, L:2.5)	11	9	11	10		3	1	3	2		34.4	2%	6%	
P4 (R:2.5, L:5)	11	11	11	11		3	3	3	3		33.1	2%	9%	
P5 (R:5, L:5)	11	11	11	11		3	3	3	3		29.3	5%	17%	
P6 (R:25, L:25)	11	11	11	11		3	3	3	3		20.2	5%	14%	
P7 (R:25, L:25)	11	11	11	11		3	3	3	3		30.7	3%	10%	
P8 (R:50, L:50)	11	11	11	11		3	3	3	3		22.5	4%	12%	
P9 (R:50, L:50)	11	11	11	10		3	3	3	2		20.9	4%	12%	

## Data Availability

Data will be accessible upon reasonable request to the corresponding author.
